# Polarographic Electrode Measures of Cerebral Tissue Oxygenation: Implications for Functional Brain Imaging

**DOI:** 10.3390/s8127649

**Published:** 2008-12-02

**Authors:** Kate Bartlett, Mohamad Saka, Myles Jones

**Affiliations:** The Centre for Signal Processing in Neuroimaging and Systems Neuroscience (SPINSN), Department of Psychology, University of Sheffield, Western Bank, Sheffield S10 2TP, UK

**Keywords:** Polarographic oxygen electrode, brain tissue, neuroimaging

## Abstract

The changes in blood flow, blood volume and oxygenation that accompany focal increases in neural activity are collectively referred to as the hemodynamic response and form the basis of non-invasive neuroimaging techniques such as blood oxygen level dependent (BOLD) functional magnetic resonance imaging. A principle factor influencing blood oxygenation, the cerebral metabolic rate of oxygen consumption is poorly understood and as such, data from imaging techniques are difficult to interpret in terms of the underlying neural activity. In particular how neurometabolic changes vary temporally, spatially and in magnitude remains uncertain. Furthermore knowledge of which aspects of neural activity are closely reflected by metabolic changes is essential for the correct interpretation of cognitive neuroscience studies in terms of information processing. Polarographic electrode measurements of cerebral tissue oxygenation in animal models following presentation of sensory stimuli have started to address these issues. Early studies demonstrated both increases and decreases in tissue oxygenation following neural activation. However a recent series of elegant studies in the cat visual system demonstrated a tight spatial and temporal coupling between evoked peri-synaptic activity and oxygen consumption following presentation of visual stimuli.

## 1. Introduction

Measurement of the partial pressure of cerebral tissue oxygen (tissue pO_2_) was first conducted in the 1950's, with Clark [1, 2] pioneering the use of insulated metal polarographic ‘cathodes’ to measure oxygen availability in the brain tissue of cats. In Clark's initial studies, changes in brain tissue pO_2_ were measured following alterations of systemic physiology. Subsequent studies examined changes in brain tissue oxygenation accompanying local increases in neural activity and found both decreases and increases in tissue oxygenation [3-5]. Recently there has been a resurgence in interest in studies investigating changes in brain tissue oxygen tension following stimulus evoked increases in neural activity and many of the questions raised by classic studies have now been addressed [6-9]. This renewed interest in evoked brain tissue oxygen changes is in part due to the popularity of modern non-invasive brain imaging techniques in humans such as positron emission tomography (PET) [10] and functional magnetic resonance imaging (fMRI) [11, 12]. These techniques rely on neurometabolic and neurovascular coupling and their associated signals rather than measuring neural activity per se, and it is hoped that an increased understanding of brain tissue oxygenation may aid interpretation of neuroimaging data in terms of clarifying the relationship between the hemodynamic response, oxygen consumption and neural activity. Key issues for the interpretation of neuroimaging data are: the spatial and temporal extent of neurometabolism; how the magnitude of neurometabolic changes relates to those of the underlying neural activity and which aspects of neural activity are faithfully reflected in neurometabolic and hemodynamic signals. Furthermore, it has recently been suggested that impaired neurovascular coupling may be a causative factor for the neurodegeneration in disorders such as Alzheimer's disease [13, 14]. Therefore data from oxygen tissue measurements that may facilitate understanding of the hemodynamic response function in terms of oxygen delivery to brain tissue, may also aid understanding of the deleterious consequences of impaired neurovascular coupling.

## 2. Brain imaging techniques rely on neurovascular and neurometabolic coupling

The resting brain requires energy, which is obtained through the process of oxidative metabolism. Despite comprising only 2% of the body's weight the brain uses 20% of the body's energy [15]. The oxygen required for metabolism is transported to brain tissues predominantly by the hemoglobin molecule found in red blood cells. During local functional activation of a brain region, local cerebral blood flow (CBF) increases above baseline, allowing delivery of freshly oxygenated blood to the active brain region. PET can provide quantitative measurements of brain hemodynamics and metabolism, which are likely to be reasonable markers of the magnitude of evoked neural activity. Depending on the tracer used different aspects of cerebral blood flow and metabolism can be measured with PET [10]. CBF can be measured by injection of oxygen-15-labeled water or carbon-11^-^labeled butanol while CBV can be measured with oxygen-15-labeled carbon monoxide or carbon-11-labelled carbon monoxide [10]. Carbon-11 or florine-18 labelled deoxy-glucose facilitates the estimation of cerebral metabolic rate of glucose (CMR_GLU_). By combining oxygen measurements from inhalation of oxygen-15-labelled molecular oxygen with those of CBF and CBV the cerebral metabolic rate of oxygen consumption (CMRO_2_) can be also estimated [16]. However, as PET requires administration of a radioactive labeled tracer this therefore makes repeated testing in the same human subject undesirable. Furthermore, due to the time required for the intra-venous tracer to reach the brain, PET has a temporal resolution of minutes and necessitates the use of block designs which makes it difficult to study the brain correlates of many psychological processes. Blood Oxygen Level Dependent (BOLD) functional Magnetic Resonance Imaging (BOLD fMRI) utilises the magnetic properties of endogenous hemoglobin to infer changes in activity in the functioning brain without the use of exogenous tracers. Depending on the number of slices taken the temporal resolution of fMRI is typically seconds or below [17] compared to the static images produced by PET.

BOLD fMRI is based on the differing magnetic properties of oxy- and deoxyhemoglobin [18]. The principle contrast agent in BOLD fMRI is paramagnetic deoxyhemoglobin, which modulates the local magnetic field to a greater extent than oxyhemoglobin. This means that protons which are nearby to deoxygenated blood easily de-phase, resulting in a decrease in magnetic resonance (MR) signal [19]. However, as the BOLD signal is predominantly based on deoxyhemoglobin, the interpretation of signal changes is complex. Deoxyhemoglobin varies with changes in all other hemodynamic parameters which are known or hypothesized to increase during activation: cerebral blood flow, cerebral blood volume (CBV), and the cerebral metabolic rate of oxygen consumption (CMRO_2_) [20]. This can make quantitative inferences of hemodynamics, and therefore neural activity from BOLD fMRI problematic.

## 3. Does oxygen consumption (CMRO_2_) increase following neural activation?

Despite the wide use of BOLD fMRI, a principal factor affecting the levels of deoxyhemoglobin during local brain activation, the local cerebral metabolic rate of oxygen consumption (CMRO_2_) is still not fully understood. In 1986, [21] Fox and Raichle contested the assumption that an active brain region requires more oxygen than when at ‘rest’. Fox and Raichle [21] obtained *in vivo* human PET measurements of local cerebral blood flow and cerebral metabolic rate of oxygen consumption at ‘rest’ and during ‘activation’ produced by somatosensory stimulation. They observed highly significant regional correlations between CBF and CMRO_2_ in the resting brain. Such correlations at rest had been observed previously by these authors [22] and in other studies [23]. However, a dramatic uncoupling of CBF and regional CMRO_2_ was observed in active brain regions following presentation of somatosensory stimuli, with 29% focal increases in CBF associated with a non-significant 5% local increase in CMRO_2_. In the visual system Fox *et al.* [24] also demonstrated an un-coupling of CBF increases (50%) and non-significant CMRO_2_ increases following presentation of visual stimuli. The metabolic rate of glucose was also measured in this subsequent study, and during activation displayed similar percentage increases to those of CBF. This led Raichle and colleagues to conclude that above baseline activity was supported by anaerobic metabolism. However, a subsequent PET study reported significant increases in CMRO_2_ following somatosensory stimulation that were approximately half those of CBF (30% versus 13%) and one PET study reported increases in CMRO_2_ during mentation that were equal to those of CBF [23]. Indeed Modern PET studies usually report robust increases in CMRO_2_ following stimulus presentation [25-27]. This has been replicated by fMRI studies making concurrent measurements of CBF (with arterial spin labeling techniques [28]) and the BOLD signal, which have also attempted to estimate local CMRO_2_ changes in following stimulus presentation [29]. These studies typically report significant robust CMRO_2_ increases [30] and CBF/CMRO_2_ ratios of ~2:1 [20, 31]. To understand why some imaging studies may have found difficulty in detecting increases in oxygen consumption despite the converging evidence suggesting the contrary, it is important to investigate stimulus evoked hemodynamics and oxidative metabolism on a finer temporal and spatial scale.

## 4. Optical techniques ‘shed-light’ on the stimulus-evoked hemodynamic response function

Indeed, at a similar time to the publication of the Fox and Raichle PET work, a technique was being implemented called ‘optical imaging of intrinsic signals’ [32] which allowed high resolution functional mapping of the cerebral cortex based on the reflectance changes that accompany neural activation [33]. In an optical imaging experiment the cortex is monochromatically illuminated and reflectance changes are measured with photodiodes or more recently CCD cameras. Depending on the frame rate of the camera it is possible to acquire data at greater at 1-2 frames per second or above. Changes in the intensity of grey level images of the cortex are due to changes in the concentration of chromophores or light scattering of brain tissue following neural activation. The principle chromophore in the brain is hemoglobin and in addition to having different magnetic properties due to its oxygenation, oxygenated and deoxygenated hemoglobin have different optical absorption spectra [34]. The choice of incident illumination can bias the origin of the intrinsic optical signal towards oxygenated or deoxygenated hemoglobin, the use of red light (600-630nm) allowing changes predominantly associated with deoxygenated blood to be examined. Optical imaging of intrinsic signals with these visible ‘red’ wavelengths, revealed a cortical ‘darkening’ (decrease in reflectance) following stimulus presentation [35], which implied an increase in deoxygenated hemoglobin. This increase in deoxygenated blood could possibly be due to a significant increase in oxygen consumption, which again is in contrast to the findings of Raichle and colleagues [21, 24]. Although the choice of wavelength of incident illumination can bias the ‘optical imaging signal’ ‘toward’ a certain chromophore, it cannot provide an absolute measure ‘uncontaminated’ by other chromophores (see Sheth *et al.* [36], for an empirical demonstration). Subsequent studies obtaining optical measures at multiple visible wavelengths using spectrographic techniques. This allowed quantitative measurements of changes in cortical hemoglobin concentration and oxygenation to be made [37, 38]. Indeed, as suggested by previous ‘single wavelength’ optical imaging studies [35] there was a transient increase in deoxyhemoglobin at stimulus onset, possibly suggesting an increase in CMRO_2_. Corresponding local cerebral blood flow responses were measured by concurrent laser Doppler Flowmetry [39, 40] and confirmed that local increases in neural activity were accompanied by increases in cerebral blood flow, which produced increases in total hemoglobin concentration (Hbt, similar to cerebral blood volume, but with the assumption of constant hematocrit). After the transient increase in deoxyhemoglobin increases in cerebral blood flow appeared to ‘outstrip’ those of cerebral oxygen consumption as CBF increases produced an increase in the concentration of oxyhemoglobin and ‘a washing away’ of deoxyhemoglobin [39, 40].

## 5. Relationship between the hemodynamic response function and the BOLD fMRI signal

The ‘washing away’ of deoxygenated hemoglobin as local CBF rises produces the so called ‘positive BOLD’ fMRI signal (a signal increase) which is most commonly used to map the locus of evoked neural activity in human neuroimaging studies. Local neural activity produces release of vasodilatory signals possibly mediated by astrocytes that dilate nearby arterioles resulting in elevated CBF. Deoxygenated hemoglobin (Hbr) is paramagnetic and increases magnetic susceptibility thus attenuating the T2* weighted MR signal. Therefore the ‘washing away’ of Hbr produces an increase in signal (a ‘brightening’ of the MR image). A less commonly used fMRI mapping signal is produced by the transient increase in deoxygenated hemoglobin immediately following stimulation which is characterised by a small signal decrease and is therefore often referred to as the ‘initial dip’, This small decrease in signal predicted by optical imaging studies was detected in some fMRI investigations [41-44]. The ‘initial dip’ was of interest to brain imaging because it was hypothesised that it may offer greater spatial concordance with the underlying neural activity than the later positive BOLD response. In the visual cortex of the cat, Malonek and Grinvald [37] demonstrated that the early increases in deoxyhemoglobin were localised to specific cortical orientation columns whereas the later washing away of deoxyhemoglobin was evident across the entire visual cortex. Indeed subsequent BOLD fMRI studies demonstrated that the earlier initial negative BOLD response could accurately map individual columns whereas the positive BOLD response could not [42].

However, the initial dip is not always detected in all studies (see [45] for review). In rodent primary somatosensory cortex, [46] optical imaging spectroscopy and oxygen-dependent phosphorescence quenching, revealed no increase in deoxyhemoglobin in response to sensory stimuli. However, Jones *et al.* [39] using the same animal model as Lindauer *et al.* [46] (the whisker barrel cortex), did observe a small increase in deoxyhemoglobin following stimulation, replicating Malonek and Grinvald's findings. The initial dip has been observed in some MR studies in human [44, 47-49], cat [42] and monkey [43] but is not always found [50]. Even when the ‘initial dip’ is detected there is also the question as to whether this aspect of the hemodynamic response is evidence for significant increases in CMRO_2_. The transient increase in deoxyhemoglobin was originally hypothesised to reflect an increase in oxygen consumption before such time as the CBF response had increased sufficiently to outstrip the demand [37]. However, Hathout *et al.* [51] modeled the system using physiologically plausible values and showed that the early deoxygenation increase could be the result of increases in regional blood flow and volume even if oxygen consumption remains constant. However, it is of note that findings suggest a corresponding decrease in oxyhemoglobin is seldom observed during the time period where deoxyhemoglobin is increasing ([52] is an exception). This led Buxton [45] to reason that both blood volume and oxygen extraction must be increasing during the period of the initial dip. If oxygen extraction alone was increasing then deoxyhemoglobin would increase and oxyhemoglobin would decrease. If volume alone was increasing then both deoxyhemoglobin and oxyhemoglobin would increase.

## 6. Measurements of brain tissue oxygenation with polarographic electrodes

Given the difficulties in analyzing optical measures of deoxyhemoglobin [46] and the suggestion that even when deoxygenation is detected it may not truly reflect increases in oxygen consumption [51], a more direct measure of the time course of *in vivo* brain oxygenation would be desirable to understand the time course of the hemodynamic response. Indeed direct measurements of brain tissue pO_2_ using fine resolution electrodes offer high temporal and spatial resolution measurements of changes in brain tissue oxygenation, and therefore are a well suited technique to address issues of neurometabolic coupling germane to the interpretation of neuroimaging data [53]. Indeed, electrode measurements of brain tissue oxygen have been made for over half a century. The amount of oxygen present in tissue is measured as partial pressure, and thus reported in units of millimeters of mercury (mmHg). The partial pressure of tissue oxygen (tissue pO_2_) can be measured using various techniques. Clark and colleagues [1, 2] were the first to use metal polarographic cathodes implanted into the brains of cats to continuously measure oxygen availability in brain tissue of unanaesthetised animals. When using polarographic cathodes, oxygen around the tip of the sensor diffuses across a membrane and is reduced at the cathode, producing a current which is proportional to the amount of oxygen available. More oxygen present in the tissue results in more oxygen diffusing through the membrane thus producing a higher current. The polarographic oxygen electrode measures available oxygen in the extracellular space which may decrease due to increased oxygen consumption or increase if delivery exceeds demand, for instance if cerebral blood flow increases. Unlike the ‘snapshots’ of cerebral hemodynamics provided by PET and MR methods, oxygen electrodes allow continuous analogue measurements which are delayed by a time constant (~300ms depending on equipment used). The spatial extent of the oxygen measurements is dependent of the tip size of the oxygen electrode with the recording site being a sphere approximately twice the tip size in diameter. Clark implanted 1-8 electrodes in the brains of cats, fixing them in place thus allowing continuous recording over time periods of between six months to over two years. Clark observed cyclic fluctuations in oxygen availability, bearing no resemblance to fluctuations in respiration, heart-rate or blood pressure. Inhalation of 100% oxygen increased the oxygen availability two-fold, whilst inducing temporary anoxia with inhalation of pure nitrogen caused a drop in oxygen availability to near zero. In the 1970's Metzger *et al.* extended Clark's studies of the 1950's, using platinum microelectrodes. In 1971, Metzger *et al.* [54] conducted experiments recording alterations in brain tissue pO_2_ following artificially induced changes in the fraction of inspired carbon dioxide. They observed linear relationships between brain pO_2_ and the concentration of inspired CO_2_.

## 7. Measurement of tissue oxygenation: stimulation induces varying types of brain oxygen responses

**Figure 1.**
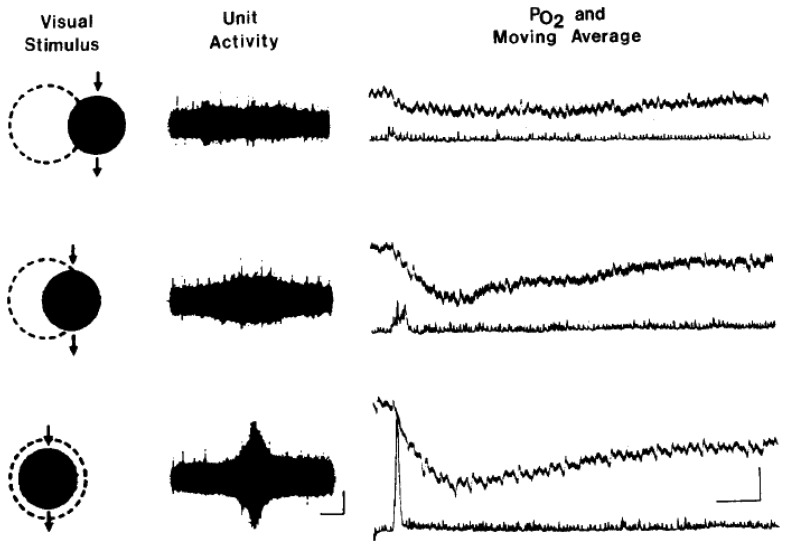
Changes in unit activity and local tissue oxygen tension in the bullfrog optic tectum following contralateral presentation of visual stimuli. Left: movement of a 10 ° black disc through different portions of the visual receptive field (approximated by broken circle). Middle: unit activity associated with each of the three stimuli. Calibration, 100/~V, 2.0 sec. Right: oxygen tension (top trace of each pair) and moving average of unit activity (bottom trace of each pair) for each stimulus: Calibration: 50 pA (3 mm Hg), 10 sec. Taken from [4] used with permission.

These electrode techniques are well suited to address the issue of whether local increases in neural activity are accompanied by significant increases in oxygen consumption, as indicated by decreases in tissue pO_2_. However, early studies investigating changes in brain pO_2_ accompanying local increases in neural activity demonstrated that both increases and decreases in tissue oxygenation could be detected. Travis & Clark [5] chronically implanted 3.3 mm diameter platinum cathodes into several cortical and sub-cortical regions in the cat brain, including the amygdala, dorsal hippocampus and medial hypothalamus and recorded tissue oxygen responses to auditory and visual stimuli. The amplitude of oxygen responses varied across brain regions, though increases in oxygenation following stimulus presentation were most commonly observed. In contrast, Sick & Kreisman [4] using very fine electrodes (1μm), reported prominent decreases in oxygen tension in bullfrog optic tectum in response to visual stimulation (Figure 1).

In this study, concurrent measures of increases in neural activity were made, implying an increase in oxygen consumption as a result of functional activation. Gijsbers & Melzack [3] recorded changes in tissue pO_2_ from the lateral geniculate nucleus (‘the visual thalamus’) of awake and anaesthetised cats in response to visual stimulation with 0.5mm diameter electrodes. Their data show that the time course of tissue oxygen changes can display variability even within a single study, as negative, positive and null oxygenation changes were observed in response to stimulation. Buerk *et al.* [55] also reported both increases and decreases in tissue oxygenation in cat optic nerve in response to visual stimulation measured with 100-150μm tip electrodes, though an oxygen decrease was more commonly observed. Even following direct electrical stimulation of the cortex both decreases [56, 57] and increases in local tissue oxygenation [53, 54] were reported. More recent studies in awake animals using more resilient carbon paste electrodes have suggested activation induced increases in brain tissue pO_2_. Carbon paste electrodes are made from Teflon-coated silver wire. The coating is slid along the wire to create a cavity which is packed with carbon paste made from a mixture of conducting graphite powder and a pasting liquid (e.g. silicone oil). A small gold electrical contact is attached to the end of the silver wire (see [58]). Carbon paste electrodes provided measures of tissue oxygen and cerebral blood flow in the striatum of freely moving rats [59]. Stimuli were a five minute tail pinch produced by attachment of a paperclip. This induced grooming in the animal, which included licking, gnawing, eating, and an overall increase in motor activity. The tail pinch resulted in immediate increase in striatal CBF which was accompanied by an increase in tissue oxygen [59]. Voltametry measurements of striatal glucose were made in a separate group of animals during an identical paradigm [59]. Decreases in extracellular glucose suggestive of in increased CMR_GLU_, were found following motor activity. This study seemed to agree to suggest an excess of oxygen delivery compared to a detectable increase in glucose use oxygen [59].

More recent studies have started to observe biphasic changes in brain tissue pO_2_ that seem to mirror those of deoxyhemoglobin. Ances *et al.* [60] made tissue pO_2_ and laser Doppler measurements of CBF changes from rat somatosensory cortex following electrical forepaw stimulation. Stimulation was at 5Hz, and both long (1 minute) and short (4 seconds) duration stimuli were used. An oxygen microelectrode was inserted 200-400μm into the cortex, and data were averaged across depths. In response to both stimulation durations, an increase in CBF preceded a tissue pO_2_ increase. However, there was an initial decrease in tissue pO_2_ from baseline, occurring <2seconds following stimulation, with the maximum decrease occurring ~0.7s after stimulation onset, preceding the increase in CBF. Masamoto *et al.* [61] also observed an early decrease in tissue pO_2_ followed by an increase in guinea pig primary auditory cortex in response to acoustic stimuli. Biphasic changes in tissue oxygenation have also been reported in rodent cerebellum following stimulation of the incoming climbing fibres [62]. However, monophasic increases in tissue pO_2_ are still reported in some studies in rat somatosensory cortex unless pharmacological interventions prevent the accompanying increases in CBF [63, 64].

## 8. How are tissue oxygen signals spatially related to the underlying neural activity?

In the most recent research, tissue pO_2_ is commonly measured by ‘Clark-style’ polarographic oxygen electrodes. As technology has advanced, the temporal and spatial resolutions of the oxygen probe have greatly increased. Modern sensors are high performance micro-electrodes. They are often glass-cased sensors, and can have a tip diameter as small in size as 7μm and a 90% response time as fast as <0.3seconds (Unisense, Denmark). These specifications make the high performance sensors ideal for obtaining rapid measurements of cortical tissue oxygenation following stimulation. A recent series of elegant of studies manipulating the spatial extent of sensory-evoked neural activity and measuring resultant oxygen tissue changes at a fine spatial scale have finally started to elucidate the factors that may result in the degree of deoxygenation and hyperoxygenation [6-9]. Indeed a key issue for the interpretation of neuroimaging signals is the spatial extent (‘point-spread’ function) of neurometabolic changes [65]. Using optical imaging spectroscopy, Malonek & Grinvald [37] were able to map individual orientation columns in the visual cortex with greater spatial precision using the early deoxyhemoglobin response than with the later washing away of deoxyhemoglobin. Thompson *et al.* [7] confirmed that this finding was due to highly focal increases in cortical activity and oxygen consumption by simultaneously measuring tissue pO_2_ and neural activity in cat visual cortex. The combined oxygen sensor and electrode had a tip diameter of 30μm thus the field of sensitivity of the oxygen sensor was a sphere approximately 60μm in diameter. This is almost an order of magnitude less than the diameter of a typical cortical column in cat (300μm to 600μm). They recorded local pO_2_ changes and spiking rate during presentation of visual stimuli of varying orientation conditions. Visual stimuli induced biphasic changes in tissue oxygenation: a decrease followed by increase. Within the primary visual cortex a particular location (‘an orientation column’) is preferentially tuned to a particular orientation of stimulus. Optimal orientation conditions elicited the largest neural responses and the most prominent decreases in tissue oxygen. As the orientation of the stimulus became less optimal for that particular location, the oxygen response displayed smaller decreases and larger increases (‘hyperoxygenation’) in pO_2_. This supported the notion that there is an increase in oxygen consumption following stimulation, which occurs on a fine spatial scale, in this case at the individual cortical column as originally suggested by Malonek & Grinvald [37].

**Figure 2.**
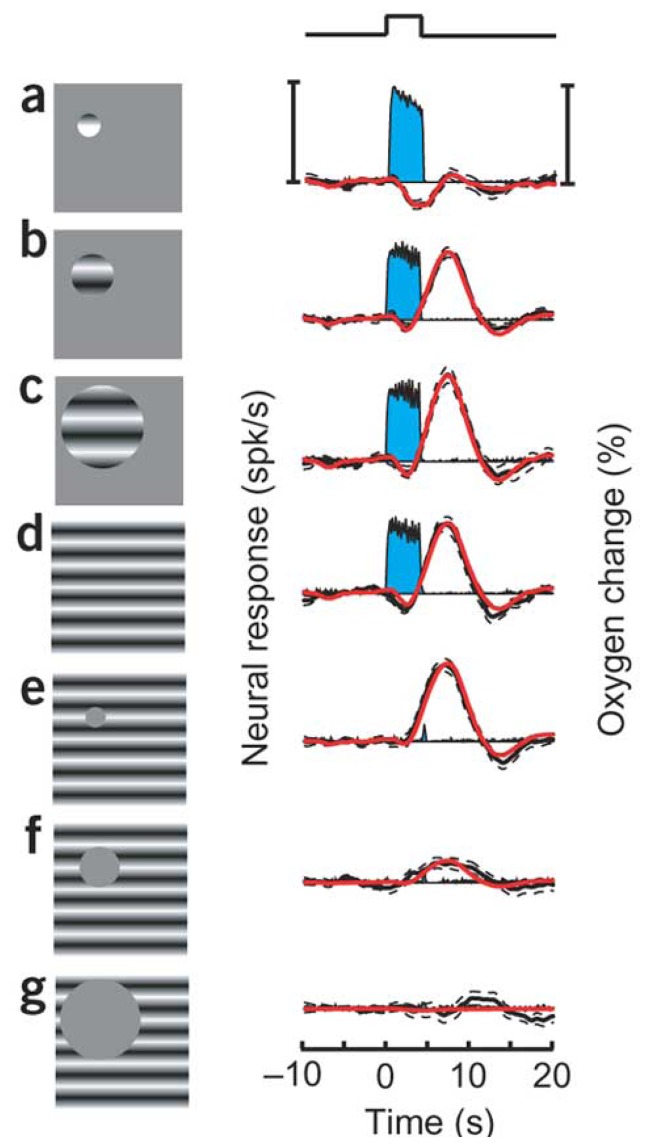
Tissue oxygen and neural responses recorded from the lateral geniculate nucleus of anaesthetised cat. Spatial extent of visual stimuli is manipulated (left paned). Negative oxygen responses are elicited by small visual stimuli in retinotopic register with electrode placement **(A).** Increases in the spatial size of the stimulus produces increases in oxygenation by activating regions neighbouring the electrode **(B-C).** This can also be achieved by ‘masking’ the retinotopic location of the electrode and not eliciting neural activity **(E-F).** Scale bars show 34 spikes per second (left) and 17% change in tissue oxygen (right). Taken from [8] used with permission.

Thompson and colleagues [8, 9] further investigated the spatial scale of brain tissue oxygenation changes following presentation of visual stimuli by exploiting the retinotopic organisation of an ‘earlier’ part of the cat visual system (the lateral geniculate nucleus, LGN, the visual area of the thalamus). Decreases in tissue pO_2_ occurred when brain activity was elicited in the region of the recording electrode whilst varying degrees of hyperoxygenation could be observed by activating neighboring regions [8] (Figure 2). Stimuli occupying a small region of retinotopic space corresponding to the topographic location of the recording electrode elicited larger neural responses and monophasic decreases in oxygen tension. Conversely, the tissue oxygen response to stimuli occupying a larger region of retinotopic space corresponding to regions outside the topographic location of the electrode showed only a small decrease in tissue pO_2_ followed by a pronounced delayed hyperoxygenation. This further suggests that decreases in tissue pO_2_ are tightly spatially coupled to neural activity, and that neural activation of neighboring regions could produce hyperoxygenation due to a coarser regulation of CBF. The spatial specificity of the oxygen response in the LGN was further investigated by changing the retinotopic distance of a ‘small’ stimulus from the topographic location of the electrode in the brain tissue [9]. It was observed that as the distance of the stimuli from the retinotopic receptive field increased the magnitude of the evoked tissue oxygen decreases became smaller, demonstrating the closely spatially localised oxygen response to the site of neural activity.

Based on these data, Thompson and colleagues [9] proposed a linear model for changes in tissue oxygenation, whereby the time course of the oxygen response is the sum of both negative and positive oxygen response components produced by neural activity both at the location of the electrode and surrounding regions. The model also incorporates the placement of the sensor in the brain in 3-dimensional space. This suggests that it is important to consider the spatial relationship of the electrode in terms of the spatial extent of evoked neural activity when interpreting the accompanying evoked tissue oxygen responses. These carefully conducted investigations may explain the previous conflicting reports of both increases and decreases in oxygen tension following sensory stimuli. In previous studies electrodes were placed in topographically ordered brain regions and stimuli were presented that may activate varying spatial extents of neural activity compared to the location of the electrode [3, 59]. Indeed when visual stimuli were presented that elicited large changes in multi-unit spiking activity (MUA), therefore confirming electrode placement in retinotopic register with the stimulus, decreases in tectal oxygen tension were reported [4]. A seminal study with direct electrical stimulation of neural tissue suggested that decreases in oxygen tension were apparent when the stimulating electrode was in the proximity of the oxygen electrode. Whereas increases in tissue oxygen could be produced if the oxygen electrode was further away from the stimulation site [57]. These studies highlight why classic PET studies averaging over a large voxel size with limited temporal resolution [21, 24] may have found difficulty in detecting significant CMRO_2_ increases, as it appears that oxygen metabolism and blood flow increases are occurring at different spatial and temporal scales.

## 9. How do the magnitudes of neurometabolic signals relate to those of the underlying neural activity?

**Figure 3.**
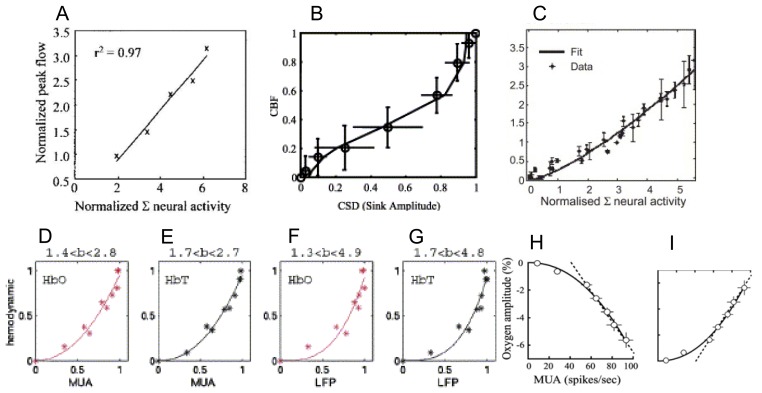
Non-linear relationships between stimulus-evoked neural activity, hemodynamics and metabolism. Both linear and non-linear relationships between neural activity and cerebral blood flow have been were reported (**A** taken from [66] used with permission, **B** taken from [67]). A subsequent study varied several stimulus parameters found that a power-law was most appropriate (**C** Taken from [68] used with permission) A power law relationship was also found to describe **(D-G)** the relationship between sensory-evoked neural activity and other hemodynamics (total hemoglobin concentration (Hbt) and oxyhemoglobin concentration (HbO_2_)) as measured by optical imaging spectroscopy) in rodent whisker barrel cortex (taken from [52] used with permission.) Despite differing animal models a similar relationship was found between neural activity and decreases in oxygen tension **(G)**. The figure is inverted to allow comparison with hemodynamics **(I).** Taken from [6] used with permission.

Following the investigations into the spatial relationship between neural activity and oxygenation, a further issue for the quantitative interpretation of neuroimaging data is how the magnitude of stimulus-evoked neural activity is related to the magnitude of changes in hemodynamics. Studies making direct quantitative measures of hemodynamic and neural signals using combined optical and electrophysiological techniques in rodent somatosensory cortex have begun to describe the relationship between brief evoked neural activity and the accompanying blood changes. Such investigations typically examine the neurovascular-coupling relationship across a range of magnitudes of sensory-evoked activity produced by varying stimulus parameters and report both linear and nonlinear relationships. Over the range of magnitudes of evoked activity produced by varying the frequency of electrical stimuli trains applied to the contralateral whisker pad, a linear relationship between neural activity and hemodynamics was reported [66]. However, in the same laboratory Jones *et al.* [67] found a non-linear relationship (an inverse sigmoid function) between increases in CBF and a range of evoked cortical neural activity produced by varying stimulus intensity (0 to 1.6mA in 0.2mA increments). The conflicting results of the two studies were reconciled by a third study in the Sheffield laboratory where both frequency and intensity of stimuli applied to the rat whisker pad were varied [68]. Over the wide range of evoked activities produced by varying both stimulus parameters, a power law function best described the relation between neural activity and CBF [68]. Over the mid-range of the magnitudes evoked neural changes (similar to those in the Martindale *et al.* [66] study), a linear relationship adequately fits the data. An inverse sigmoid function could fit the small number of data points created by varying stimulus intensity alone. This study suggested that a ‘power law’ is the most appropriate to capture a large number of evoked activities and accompanying CBF responses. Indeed Sheth *et al.* [69] also reported a non-linear power law relationship between evoked activity and hemodynamics in rat somatosensory cortex by producing a range of evoked neural activity by varying the amplitude and frequency of electrical hindpaw stimulation. They concluded like Hewson-stoate *et al.* [68], that when a limited range of data is present, a linear relationship may be observed, but that this may be an incomplete and inaccurate conclusion when more stimulus conditions are included. Devor *et al.* [52] also observed a non-linear power law relationship between evoked neural activity and hemodynamics following stimuli applied to a single whisker rather than the entire whisker pad suggesting this relationship is appropriate even at the level of an individual whisker barrel column (Figure 3 A-D).

The question remained as to whether this non-linearity was due to neurometabolic or neurovascular coupling. A subsequent study in the Berkeley laboratory [6] measured pO_2_ responses in cat LGN to visual ‘checkerboard’ stimuli. The contrast of the checkerboard stimuli was altered to elicit a range of sensory-evoked activities. As for the hemodynamics data the relationship between evoked neural activity and decreases in tissue pO_2_ was best described by a non-linear power law. This suggests that the relationship between the magnitudes of hemodynamics and neural activity is comparable to those of neural activity and oxygen metabolism (Figure 3).

## 10. How do the temporal dynamics of neurometabolic signals relate to those of the underlying neural activity?

Although studies examining the magnitudes of evoked hemodynamics, metabolism and the magnitudes of the underlying evoked activity can aid the interpretation of neuroimaging studies where sensory stimuli are presented [70, 71] in more complex cognitive neuroscience experiments the duration of neural activity elicited may be unknown or vary between brain regions. Therefore it is important to understand to the relationship of the entire time series of hemodynamics and metabolic changes to those of the underlying neural activity. Martindale *et al.* [66] found that the hemodynamic response to a brief stimulus (2s) of the rat whisker barrel cortex can be accurately modeled using a linear convolution of a hemodynamic impulse response function and the neural events in response to an electrical stimulus pulse train applied to the whisker pad. However, this ‘linear-convolution model’ could not predict the CBF responses to stimuli of a longer duration (8s, 16s duration [72]). This finding was previously reported by Ances *et al.* [73] who found that a linear time invariant model could not predict CBF changes from those of neural activity when stimulus duration was greater than 4s.

**Figure 4.**
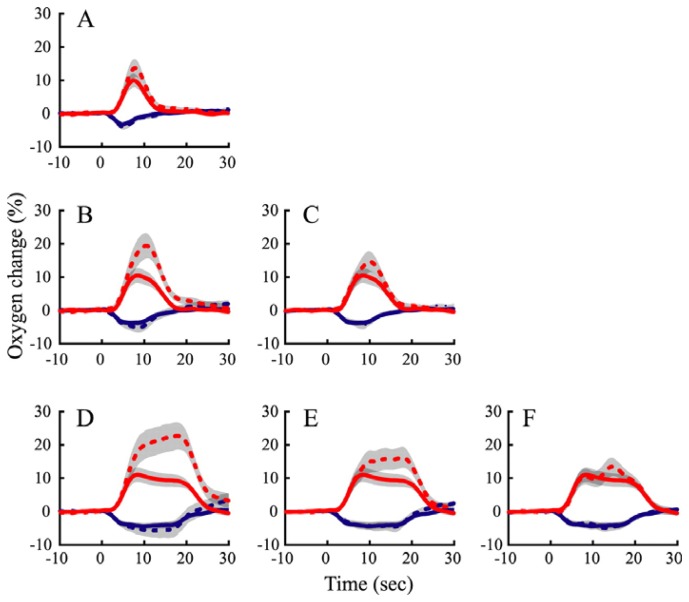
Time-invariant analysis of negative and positive oxygen responses in cat lateral geniculate nucleus. Predicted responses to long-duration stimuli were generated by replicating, shifting, and summing measured responses to short-duration stimuli. Oxygen responses to four stimulus durations are used to make six predictions. (***A)*** Measured (solid curves) and predicted (dashed curves) oxygen responses to 4 s stimuli based on responses to 2 s stimuli. Blue and red curves represent oxygen responses to the small (negative) and large (positive) visual stimuli, respectively. Gray filled areas represent ± 1 SE. *B*, *C*, Measured and predicted oxygen responses to 8 s stimuli based on responses to 2 s **(*B*)** and 4 s **(*C*)** stimuli. ***D–F***, Measured and predicted oxygen responses to 16 s stimuli based on responses to 2 s **(*D*),** 4 s **(*E*),** and 8 s **(*F*)** stimuli. Taken from [6] used with permission.

The question again remained as to whether these nonlinearities were due to neurometabolic or neurovascular coupling. To address this issue the duration of visual stimuli were varied and resultant measured evoked changes in neural activity and tissue oxygen in cat LGN were measured [6]. As in previous studies [8, 9] these authors also varied the spatial extent of evoked neural activity by varying the ‘size’ of visual stimuli presented. Again when the stimulus occupied a small region of retinotopic space corresponding to electrode location, this produced decreases in tissue oxygen, whereas when the stimulus occupied a larger region of retinotopic space corresponding to neighboring regions, there were increases in tissue oxygen. Li and Freeman investigated whether tissue oxygen responses to longer duration stimuli could be estimated by convolving the responses to shorter duration stimuli with a stimulus pulse function (Figure 4).

With regard to the positive oxygen responses produced by large visual stimuli, the convolution model over-predicted the long duration responses, similar to those often reported for hemodynamics [72, 73]. However, a linear convolution model was able to fit the negative oxygen changes, with responses to short duration stimuli accurately predicting the long duration data. The negative tissue oxygen responses occur when the stimuli are activating a more specific, localised retinotopic space, as opposed to the positive tissue oxygen changes, which occur in response stimuli activating larger retinotopic regions. This indicates that the nonlinearities in temporal neurovascular coupling are introduced by hemodynamics, and not metabolism, and suggests that the highly localised neural activity and tissue oxygen decrease elicited by the spatially more specific visual stimulus are closely coupled in time as well as space.

## 11. Which aspects of neural activity are most related to neurometabolic signals?

The final issue for the interpretation of brain imaging techniques is which aspects of evoked neural activity are most accurately reflected in the neurometabolic signal. With regard to measurements of neural activity, local field potentials (LFP) reflect synaptic activity and the synchronised input to the activated region and intra-regional processing [74] while multi-unit activity (MUA) represents the spiking output of neurons in the active region [74]. In simple experimental paradigms such as those used to investigate neurovascular coupling in somatosensory cortex the relationship between sensory-evoked LFP and MUA is close to linear [52, 67]. However, in more complex cognitive tasks dissociations between LFP and MUA can occur [75] and the two may convey different aspects of information processing [76], making it important to know which one can be inferred from imaging techniques like fMRI. However, in commonly used cortical somatosensory animal models of neurovascular coupling it is difficult to dissociate LFP and MUA [52, 67] making them unable to address this issue.

However in some brain systems careful manipulation of stimulus parameters may enable dissociation of evoked MUA and LFP, allowing the accompanying metabolic changes to be examined. Although, previous studies in the Berkley laboratory used MUA alone as a metric of spatially localised activity [6-9] a subsequent study measured both LFP and MUA to attempt to investigate which of these measures was most related to the neurometabolic signals assessed by oxygen sensors. Viswanathan & Freeman [77] presented visual stimuli at varying temporal frequency (‘flicker rate’) and measured tissue oxygen responses and neural activity in the visual cortex of anaesthetised cats. The visual cortex only responds to low temporal frequency stimuli while the LGN (an earlier stage of visual processes that projects directly to cortex) can respond to both low and high temporal frequency visual stimuli. Therefore high temporal frequency stimuli produce LFP but not spiking responses in V1, whereas low temporal frequency stimuli produce both LFP and MUA responses. They showed that tissue pO_2_ changes can occur in the absence of local spiking activity, as tissue oxygen responses were present in both low and high frequency conditions, whereas MUA responses were only observed in response to low temporal frequency stimuli. As in their previous studies which made recordings in LGN, varying the spatial extent of the stimulus presented allowed the authors to manipulate the degree of hyperoxygenation and deoxygenation detected by the electrode (this time in cortex). This enabled demonstration that both the positive and negative changes in tissue oxygen elicited by visual stimuli were present in both temporal frequency conditions and therefore in the absence of spiking. However, some investigators have raised question with the study, suggesting that the evidence for decreases in tissue oxygenation in the absence of spiking activity was not as convincing those for the positive responses [78]. There is converging evidence that LFP rather than MUA is a better correlate of neuroimaging signals, but all these studies involve positive BOLD signals or CBF increases, which are presumably more related to tissue hyperoxygenation than deoxygenation. For instance unpublished observations from the Logothetis laboratory suggest that visual cortical positive BOLD fMRI responses can be elicited by both low and high temporal frequency stimuli [53]. Simultaneous recordings of neural activity and positive BOLD fMRI signals in anaesthetised primate also suggested that local field potentials (LFP), were better predictors of the BOLD response than single and multi-unit activity (MUA) [79]. The MUA increase in response to visual stimuli was transient, rapidly returning to baseline, thus showing adaptation to the stimulus throughout its presentation. However, the increase in LFP remained throughout the duration of the stimulus, similar to the time course of the BOLD fMRI signal. This finding was recently replicated in awake behaving primates [80]. Further evidence for the role of peri-synaptic rather than spiking activity giving rise to neuroimaging signals is provided by another simultaneous fMRI and electrophysiological study in primate [81]. Following direct microinjection of a 5HT-1A agonist into the primary visual cortex, visual stimuli evoked LFP responses while MUA responses could no longer be recorded. However, accompanying positive BOLD fMRI responses were still evident. Furthermore, in the cerebellum, where it is possible to produce dissociations between field potentials and spike activity by simultaneous climbing and parallel fiber stimulation, it has been shown that CBF correlates with field potential even in the absence of spiking output [82]. Although there is considerable evidence that LFP is more related to neuroimaging signals than MUA most studies have focused on positive BOLD signals or CBF increases, it raises the possibility that different these aspects of neural activity could be related to different aspects of neurometabolic and neurovascular responses.

## 12. Neurovascular coupling and tissue oxygenation across the cortical laminae

Polarographic electrode measurements of tissue oxygenation responses to sensory stimuli have furthered understanding of the spatial and temporal extent of neurometabolic signals and to which aspects of information processing they relate. However, a particular issue for the interpretation of neuroimaging signals essential for the understanding the cortical processing, is how neurovascular coupling varies as a function of cortical depth. In addition to its columnar organisation perpendicular to its surface, the cortex is a complex layered structure with inputs and outputs at distinct laminae, an understanding of which is also vital to the appreciation of how the cortex processes information [83]. An appreciation of the relative oxidative demands at different depths could inform the interpretation of multi-laminar fMRI studies [84] and the fine spatial resolution of polarographic oxygen electrodes seems ideally suited to address this issue. Furthermore, such data could further elucidate the relative contributions of spiking and synaptic activity to neurometabolic signals, as it has been observed that these different types of evoked neural activity changes display differing profiles across the cortical depth [67]. Indeed, differences in baseline oxygen tension at different depths have long been demonstrated using this technique. As a polarographic microelectrode is advanced down through the cortex tissue pO_2_ shows a decrease as depth into the cortex increases [85, 86]. However, studies have seldom examined stimulus-evoked tissue oxygen changes at different cortical depths. LaManna *et al.* [87] observed differences in tissue oxygen response at varying cortical depths produced by direct electrical stimulation of the cortex, but it is difficult to know whether sensory stimuli would produce similar findings. The data of Masamoto *et al.* [61] may suggest differing tissue pO_2_ responses through the cortical laminae, as a negative tissue pO_2_ response in guinea pig auditory cortex was shown to be more prominent at superficial depths than the hyperoxygenation response. However, their data were obtained from only a single experimental subject. Differences in evoked tissue oxygen responses have also been recently observed at different laminae of the olfactory bulb [88]. Further advances may come from carrying out laminar investigations in a more commonly used animal model of neurovascular coupling such as the whisker barrel cortex. This provides an excellent animal model for potential investigation into a depth profile of tissue oxygenation due to its highly organised topographic organisation in the primary somatosensory cortex [89]. Indeed, a recent preliminary report suggests monophasic negative responses at cortical depths associated with the greatest magnitude of whisker-evoked LFPs [90].

## 13. Summary

Polarographic electrode studies of brain tissue oxygenation have aided the understanding of metabolic changes induced by focal increases in neural activity. Although initial studies reported both decreases and increases in brain tissue oxygenation following stimulus presentation, modern studies in the visual system of cats have managed to elucidate some of the factors contributing to the degree of deoxygenation and hyperoxygenation observed. With brief stimuli the magnitudes of evoked neural activity are related to those of oxygen metabolism by a non-linear power law, a similar relationship to that reported between neural activity and hemodynamics. With regard to the temporal dynamics of negative oxygen changes and neural activity, linear convolution models can be used to accurately predict the responses to longer stimuli from shorter ones. Temporal nonlinearities only occur for positive oxygenation responses similar to those reported for hemodynamics. Varying stimulus parameters to produce dissociations between evoked synaptic activity (LFP) and local spiking (MUA) has demonstrated that neurometabolic responses are more related to the peri-synaptic processes reflected by local field potentials. The post-synaptic targets of pre-synaptic glutamate release include glial cells which can initiate arteriolar relaxation and the well characterised hemodynamic response. This release also produces fluctuations in the membrane potentials that give rise to local field potentials.[91]. Further studies investigating laminar profiles of evoked cortical tissue oxygen responses from multiple cortical depths may further advance our understanding of neurometabolic coupling in terms of cortical processing.
